# Evaluation of models for estimation of genetic parameters for post-weaning body measurements and their association with yearling weight in Nellore sheep

**DOI:** 10.5713/ab.20.0426

**Published:** 2024-02-22

**Authors:** Satish Kumar Illa, Gangaraju Gollamoori, Sapna Nath

**Affiliations:** 1 Livestock Research Station, Sri Venkateswara Veterinary University, Garividi, Andhra Pradesh State 535101, India; 2 College of Veterinary Science, Sri Venkateswara Veterinary University, Tirupati, Andhra Pradesh State 517502, India; 3 Sri Venkateswara Veterinary University, Livestock Research Station, Garividi, Andhra Pradesh State 535101, India

**Keywords:** Body Measurements, Heritability, Nellore Sheep, Restricted Maximum Likelihood (REML)

## Abstract

**Objective:**

The objective of this study was to obtain (co) variance components and genetic parameter estimates for post-weaning body measurements such as body length (BL), height at withers (HW), and chest girth (HG) recorded at six (SBL, SHW, and SHG), nine (NBL, NHW, and NHG) and twelve (YBL, YHW, and YHG) months of age along with yearling weight (YW) in Nellore sheep maintained at livestock research station, Palamaner, Andhra Pradesh, India and also the association among body measurements with YW was studied.

**Methods:**

Data on 2,076 Nellore sheep (descended from 75 sires and 522 dams) recorded between 2007 and 2016 (10 years) were utilized in the study. Lambing year, sex of lamb, season of lambing and parity of dam were included in the model as fixed effects and ewe weight was kept as a covariate. Analyses were conducted with six animal models with different combinations of direct and maternal genetic effects using restricted maximum likelihood procedure. Best model for each trait was determined based on Akaike’s information criterion.

**Results:**

Moderate estimates of direct heritability were obtained for the studied traits viz., BL (0.02 to 0.24), HW (0.31 to 0.49), and CG (0.08 to 0.35) and their corresponding maternal heritability estimates were in the range of 0.00 to 0.07 (BL), 0.13 to 0.17 (HW), and 0.07 to 0.13 (CG), respectively. Positive direct genetic and phenotypic correlations among the traits and they ranged from 0.07 (YBL-YW) to 0.99 (SBL-SHG, SHG-YW, and NBL-YBL) and 0.01 (SBL-YBL) to 0.99 (NBL-NHG), respectively. Further, the genetic correlations among all the body measurements and YW were positive and ranged from 0.07 (YW and YBL) to 0.99 (YW and SHG).

**Conclusion:**

There was a strong association of chest girth at six months with YW. Further, it is indicated that moderate improvement of post-weaning body measurements in Nellore sheep would be possible through selection.

## INTRODUCTION

Nellore is one of the recognized native sheep breeds of southern India and are reared for meat and known for its superior growth rate. This sheep is tallest and characterized by white with black spots around the lips, eyes, lower jaw and abdomen. They are predominantly distributed in south coastal districts of Andhra Pradesh state. Its population was about 11.74 million and accounts for 19.17 per cent of total sheep population of India [1]. These sheep possess better adaptability to hot and humid climatic conditions and also resistant to most of endemic infectious diseases.

In sheep meat yield is an intricate quantitative trait found to be affected by several genetic and non-genetic factors. However, body measurements or biometric traits were controlled by uncomplicated genetic mechanisms. Hence, biometric traits could be used as a selection criterion in improving the farm animals in terms of meat-yield. Biometric traits in sheep are majorly affected by individual’s own genes as well as maternal genes and maternal environment apart from the environment in which it is raised [2–4].

Genetic evaluation of sheep for economically important traits is prerequisite and plays pivotal role in formulating breeding objectives and helps in conservation of germplasm. Several investigations on growth and its related traits stated that the traits are affected by both direct additive genetic effects and maternal genetic effects and models which ignored maternal genetic effects resulted in biased estimation of genetic parameters [5–9]. To implement better breeding plans, information on (co)variance components and genetic parameters for these traits are prerequisite.

In Nellore sheep, the genetic parameters and (co)variance components for body weight, average daily gain and Kleiber ratio’s at different ages were estimated using animal model, direct genetic and maternal effects on the traits were evaluated, genetic diversity and effects of inbreeding on growth traits in Nellore sheep were also assessed in this sheep [10,11]. Rajkumar et al [12] studied the effect of management system (semi-intensive vs intensive) on the slaughter age with respect to carcass characteristics and other related traits. The study recommended the slaughter of Sirohi kids at nine months of age for those reared under intensive management system and at twelve months for those maintained under intensive system of management. Similarly, Nellore lambs attain optimum weight at yearling age, which is suitable for the slaughter. With this hypothesis, the present study was undertaken to estimate the variance components and genetic parameters for post-weaning body measurements, to estimate phenotypic, genetic and environmental correlations among the traits and also to know the association of body measurements with the yearling weight and to identify the major non-genetic factors affecting the traits.

## MATERIALS AND METHODS

### Data collection

In this study, data were obtained from the Nellore sheep flock maintained at the Livestock Research Station; Palamaner, Andhra Pradesh, India (located at 13°.20’E latitude and 78º.75’N longitude at an altitude of 683 m above mean sea level) over a period of ten years (2007 through 2016). A total of 2,076 lambs descended from 75 sires and 522 dams were considered for analysis. Different body measurements considered in the analysis were body measurements recorded at 6, 9, and 12 months of age viz., body length (BL), height at withers (HW), and chest girth (HG) and along with yearling weight of lambs and the body measurements were recorded [13].

### Animal management

All the animals in the flock were reared under semi-intensive system of management. Four hundred females were maintained in the flock with male to female ratio for breeding was around 1:25. Ten to fifteen sires were kept for breeding per year. Sires used for breeding were retained in the flock for at least two years, males were selected based on their six months weight and their progeny performance was also considered for their selection. Breeding in the flock was confined to major (March to May) and minor seasons (July to September). Selection was not practiced for ewes. Females were bred either at an age of 15 months or after attaining 25 kg body weight. Culling was done twice in a year especially at the onset of breeding season, low production and poor health status were the basis for culling. Oestrus synchronization technology covers limited number of ewes i.e., around 30 to 40 in the flock. Body measurements of new born lamb was taken within 10 hours of birth, remaining body measurements and weights were recorded at 3, 6, 9, and 12 months of age precisely on exact dates.

Weaning of lambs was done at an age of three months. Lambs were fed with concentrates *ad libitum* from ten days after birth till weaning. From six months of age, animals were sent for grazing for a period of 8 to 10 hours. In addition to this, 300 g of concentrate mixture was provided during post-weaning period. Fodder tree loppings and hay of *Stylo hamata*, cow pea, horse gram and alfalfa were also fed to animals. Seasonal differences were observed in the growth patterns of animals during March to June due to limited grazing resources. Grazing area consisted deciduous vegetation and fodder trees like Subabul (*Leucaena leucocephala*), Neem (*Azadirachta indica*), and Avisa (*Sesbania grandiflora*).

### Statistical methods

Initially data were analyzed to know the fixed effects to be included in the model by least-squares analysis of variance [14]. The model included the fixed effects of year of lambing (nine levels), season of lambing (two levels), sex of the lamb (two levels) and parity of dam (seven levels). Ewe weight at lambing was kept as a covariate. Only significant effects (p≤ 0.05) were included in the models which were subsequently used for genetic analysis. Convergence of the restricted maximum likelihood (REML) solutions was assumed when the variance of function values (−2 log-L) in the simplex was less than 10^−8^. To ensure that a global maximum was reached, the analysis was restarted. When estimates did not change up to two decimals, convergence was confirmed. Six models which accounted for the direct and maternal effects were fitted and are as follows:


model 1 $$y=\mathrm{Xb}+Z_{a}a+e$$

model 2 $$y=\mathrm{Xb}+Z_{a}a+Z_{m}m+e\;\mathrm{with}\;\mathrm{Cov}\;(a_{m},m_{o})=0$$

model 3 $$y=\mathrm{Xb}+Z_{a}a+Z_{m}m+e\;\mathrm{with}\;\mathrm{Cov}\;(a_{m},m_{o})=A\sigma_{\mathrm{am}}$$

model 4 $$y=\mathrm{Xb}+Z_{a}a+Z_{\mathrm{pe}}\mathrm{pe}+e$$

model 5 $$y=\mathrm{Xb}+Z_{a}a+Z_{m}m+Z_{\mathrm{pe}}\mathrm{pe}+e\;\mathrm{with}\;\mathrm{Cov}\;(a_{m},m_{o})=0$$

model 6 $$y=\mathrm{Xb}+Z_{a}a+Z_{m}m+Z_{\mathrm{pe}}\mathrm{pe}+e\;\mathrm{with}\;\mathrm{Cov}\;(a_{m},m_{o})=A\sigma_{\mathrm{am}}$$

Where, ***y*** is the vector of records; ***b***, ***a***, ***m***, ***pe***, and ***e*** are vectors of fixed, direct additive animal genetic, maternal additive genetic, permanent environmental effects of the dam and residual effects, respectively, with association matrices ***X***, ***Z****_a_*, ***Z****_m_*, and ***Z****_pe_*; ***a****_m_*, ***m****_o_* are the direct and maternal genetic effects, respectively. A is the numerator relationship matrix between animals; and ***σ****_am_* is the covariance between additive direct and maternal genetic effects. Assumptions for variance (V) and covariance (Cov) matrices involving random effects were


$$\begin{array}{l} \text{V}(\text{a})=\text{A}{\sigma^{2}}_{\text{a}},\;\text{V}(\text{m})=\text{A}{\sigma^{2}}_{\text{m}},\\ \text{V}(\text{pe})=\text{I}{\sigma^{2}}_{\text{pe}},\;\text{V}(ɛ)=\text{I}{\sigma^{2}}_{\text{e}},\;\text{and Cov}(\text{a},\text{m})=\text{A}\sigma_{am} \end{array}$$

Where, I represent identity matrix; σ^2^_a_, σ^2^_m_, σ^2^_pe_, and σ^2^_e_ are additive genetic variance, additive maternal, maternal permanent environmental and residual variances respectively. The direct-maternal correlation (r_am_) was obtained for all the traits under analysis. Maternal across year repeatability for ewe performance was calculated for all the traits as ${\text{t}}_{\text{m}}=(1/4)\;{\text{h}}^{2}+{\text{m}}^{2}+{\text{c}}^{2}+{\text{r}}_{\text{am}}\;\sqrt{{\text{m}}^{2}}\sqrt{{\text{h}}^{2}}$ [15]. The total heritability (h^2^_t_) was calculated using the formula: h^2^_t_ = (σ^2^_a_+0.5σ^2^_am_ +1.5σ_am_)/σp^2^ [16].

Six different models were used to estimate the (co) variance components and genetic parameters and the model with lower Akaike’s information criterion (AIC) values were chosen as the best model. Model 1 is a simple one included only animal direct genetic effects and as it did not consider maternal genetic components and yielded biased estimates. Whereas, model 2 considered both direct additive and maternal genetic effects and yielded better estimates of direct heritability. Model 3 included covariance between direct additive and maternal genetic effects. Similarly model 4 incorporated maternal permanent effects and model 5 included all the genetic and environmental effects, whereas, model 6 is a comprehensive model as it included the covariance between genetic and environmental effects.

Estimates of (co)variance components were obtained by REML using wombat software program [17]. Genetic parameters were estimated by fitting univariate animal models including and ignoring maternal effects. The AIC was computed to rank the models. If p denotes the number of random (co)variance parameters to be estimated and Log L is the maximized likelihood, then the information criterion is defined as AIC = −2 Log L+2p [18]. The model yielding the smallest AIC explains better variation in the trait. Subsequently, a series of bi-variate animal model analysis was carried out to estimate genetic and phenotypic correlations between the traits with starting values obtained from single trait analysis.

## RESULTS

Number of records, pedigree structure, summary statistics and different sources of variation for post-weaning measurements in Nellore sheep are shown in Table 1. The least-squares means obtained in our study for body lengths at 6, 9, and 12 months of age were 57.62±0.12, 59.72±0.13, and 62.51±0.13 cm, respectively. Likewise, heights at withers were 65.27±0.14, 69.73±0.13, and 74.43±0.12 cm, respectively, whereas the corresponding means for chest girth were 69.76±0.15, 70.26 ±0.13, and 75.50±0.10 cm respectively. Least-squares means for weight at 12 months of age in Nellore sheep are found to be 26.21±0.12. In general, the coefficient of variation for the studied body measurements were ranged from 6.01 to 10.81 per cent. All the fixed effects incorporated in the model were found to be significant on post-weaning body measurements with few exceptions. The coefficients of determination for fitted models were ranged from 0.44 to 0.51 per cent.

Based on the AIC values, best model explaining the source of variation for body lengths at 6, 9 and 12 months of age were 3, 2 and 2 models, respectively. Whereas, 2, 2 and 6 were observed to be best models for HW at these ages and the best model for the corresponding chest girth were 3, 4 and 6 and model 6 explained better variation for yearling.

The direct heritability estimates obtained for body measurements (SBL, SHW, and SCG) at sixth months were 0.13, 0.31, and 0.08, respectively. Heritability estimates observed at nine months of age were 0.24, 0.38, and 0.35, respectively and at yearling age the estimates observed were 0.02, 0.49, and 0.32, respectively. The corresponding estimates of maternal heritability for body measurements at six months, nine and twelve months were 0.07, 0.13, 0.07, 0.00, 0.17, 0.00, 0.20, 0.13, 0.09, respectively. Maternal permanent effects were insignificant for all the traits except in traits NHG, YHW, YHG, and YW. For yearling weight estimates of direct and maternal heritability, maternal permanent effects obtained were 0.11, 0.09, and 0.06, respectively (Table 2).

Total heritability is useful in assessing the expected response to phenotypic selection for the traits and the estimates for all the post-weaning body measurements were ranged from 0.02 to 0.47. Similarly, repeatability of ewe performance explains the total maternal and ewe transmitted effects and the estimates obtained in the present study ranged between 0.01 and 0.27.

Results from the bi-variate analysis revealed positive direct genetic correlations among the post weaning body measurements (Table 3). At six months age, the genetic correlations between the body measurements (SBL, SHW, and SHG) of Nellore sheep ranged from 0.26 to 0.99, the corresponding phenotypic and environmental correlations are in the range of 0.01 and 0.98, −0.007 and 0.98, respectively. The genetic correlations of these body measurements with yearling weight varied from 0.26 to 0.99 and the corresponding phenotypic and environmental correlations estimates are found to be 0.25, 0.27, 0.98 and −0.007, 0.007, 0.97, respectively. Whereas, the genetic correlation estimates of the body measurements at nine months age (NBL, NHW, NHG) are observed to be in the range between 0.40 to 0.99, low to high phenotypic and environmental correlations are noticed for these traits (0.11 to 0.99, 0.05 to 0.99). The genetic correlation estimates for the body measurements at yearling age (YBL, YHW, YHG) are in the range of 0.07 and 0.87, low to moderate correlations (Phenotypic and Environmental) are observed among these traits. However, the genetic, phenotypic and environmental correlation estimates of yearling body measurements with yearling body weight are found to be 0.07, 0.27, 0.87, 0.22, 0.24, 0.28 and 0.19, 0.25, 0.40, respectively.

## DISCUSSION

The overall least-squares means for the post-weaning body measurements obtained in our study were in agreement with the findings of earlier researchers in various sheep breeds [2–4]. Lower magnitude of coefficient of variation observed in the studied traits suggested that the traits were almost uniform and lowered differences among the animals and minor changes in these traits by environmental factors. Similar results were reported in the literature in various sheep breeds [3,19–22]. All the studied traits were significantly affected by year of lambing (p<0.01) and the differences could be attributed to the variations in environmental conditions such as rainfall, temperature and humidity, pasture availability, management conditions, disease outbreaks, production systems, grazing pattern, nutrition and breeding strategies prevailed during the years and this finding was in congruence with the findings of Mandal et al [2] and Bakhshalizadeh et al [4] Male lambs had higher body measurements than females and similar results were reported in Muzaffarnagari and Moghani sheep.

Differences in sexes for body measurements were due to differences in their endocrine system. In females, estrogen hormone did not support the growth of long bones, whereas the testosterone had positive effect on body measurements as it acts like growth hormone in males [19].

Parity of dam had significant effect on biometric traits and this finding was in agreement with the findings of Mandal et al [2] and Jafari et al [23]. It is obvious that primiparous ewes produce lambs with low body weight and measurements. Ewes in their early parity could not attain the adult body weight or mature weight. Hence, considerable portion of energy would be spent on body weight gain, in addition to the fetal growth. Further, it is well acclaimed that the better mothering ability is observed in ewes with higher parity order.

The direct heritability estimates obtained in the present study for body measurements of Nellore sheep at various ages were moderate and higher in magnitude. Lower estimates than the present study was reported in Muzaffarnagari sheep and the estimates for body measurements at six, nine and yearling ages were SBL (0.11), SHW (0.14), SHG (0.14), NBL (0.15), NHW (0.18), NHG (0.24), YHW (0.19), and YHG (0.24) (Mandal et al [2]). Except for body length in adults, low estimates of direct heritability were reported in Moghani sheep (height at withers [0.037], chest girth [0.073]) and these estimates were based on Gibbs sampling [24] moderate to high estimates of heritability for BL, HW, and HG (BL [0.30, 0.35 and 0.28], HW [0.43, 0.57 and 0.40] and HG [0.45, 0.39 and 0.40]) in Blue du Maine, Suffolk and Texel sheep [25], respectively was observed. Higher estimates of direct heritability (BL [0.72], HW [0.70], and HG [0.56]) than our present estimates were observed in East Friesian and Black-Brown milk sheep [26]. Low direct heritability estimates for body length than our study (0.005) reported in Santa Ines sheep [27]. Bakhshalizadeh et al [4] estimated direct heritability for HW, BL, and HG in Moghani sheep as 0.10, 0.16, and 0.11, respectively which were lower in magnitude compared to our study. Oliveira et al [28] estimated similar estimates of direct heritability for heart girth (0.25), height at withers (0.48), and body length (0.24) in Santa Ines sheep. Abbasi and Ghafouri-kesbi [29] obtained low to medium direct heritability estimates for morphometric traits in Makuie sheep. Higher estimates of direct heritability for YW in Makuie sheep (0.22 and 0.36, respectively) [3,26]. The direct heritability estimates for YW in Moghani sheep as 0.17 [24]. Similar estimate of direct heritability (0.10) for YW was reported in Barki lambs [30].

The moderate estimate of heritability for post-weaning body measurements at various ages could be attributed to the favorable conditions at grazing which minimized the residual variance and also the environmental differences among the animals were minimum leading to the better expression of animal genes. Falconer, opined that environmental variance is a property of genotype up to some extent, where certain genotypes are more sensitive to the environmental differences [31]. Hence, it is concluded that favorable environmental variations may resulted in better estimates of heritability. It is also indicated that moderate genetic progress would be expected through selection.

In the present study, very low estimates of maternal heritability were obtained. Height at withers at six months and nine months were affected significantly by maternal genetic effects with a proportion of 13 and 17 per cent to the total genetic variation. Similar results were reported in Moghani sheep and Muzaffarnagari sheep, respectively [2,4]. Low and zero estimates of maternal genetic effects for body measurements at various stages of growth suggested that maternal genetic effects were not much important in these traits. Although, the maternal heritability estimate for NBL is zero, the best model explaining the variation is found to be model 2. This could be explained by the fact that the maternal genetic variance for NBL is very less in magnitude but not zero.

Introducing a non-zero (co) variance component between direct and maternal genetic effects (model 3 and 5) generates a negative correlation between these effects. However, the negative r_am_ would not be possible from a biological perspective [32]. The probable reason for the negative r_am_ could be due to poor environmental conditions, such as udder problems, non-sufficient nutrition and experimental conditions [17]. However, studies revealed that the structure of data plays the major role in the negative correlation between direct and maternal genetic effects [3], in which a low and high number of progeny records per dam may resulted in negative and positive r_am_, respectively [32].

Model 4 and 6 included maternal permanent effects as random affects and incorporated random effects explained 4, 3, 6 and 6 per cent of total variation in NHG, YHW, YHG, and YWT, respectively. This portion of variation was found to be non-significant in all the studied traits. Individual’s own genotype is decisive than other effects for body measurements. However, certain traits like SHW, NBL, NHW, and YBL were affected by maternal genetic effects suggested that still post weaning has some carry over effect of maternal genetic effects as a source of variation. Hence, it is imperative to consider maternal effects for genetic evaluation of post weaning body measurements.

Total heritability estimates in some of the traits are slightly higher than direct heritability estimates, due to the existence of maternal genetic effect and it is not true, if the correlations are large and negative. The estimates of total heritability were observed to be low, if negative correlation exists between additive direct and maternal genetic effects and higher estimates of total heritability were observed with low r_am_ values (Table 2). This finding was in agreement with reports for Moghani and Makuie sheep [3,22]. Presence of negative (co) variance in the model reduces the total heritability estimate and in turn affects the potential response to selection [32].

In the present study, the additive genetic correlations were higher than the phenotypic correlations and they ranged from low to moderate to high in magnitude. Positive genetic correlations among the traits indicated that traits are under control of some common genes and selection for improvement of one trait will lead to the improvement of another trait. Similar to our results, positive genetic correlations between body measurements and YW in Makuei sheep was observed [3]. High and positive direct genetic correlations between body length and height at withers (0.81), body length and height at girth (0.82) and height at withers and heart girth (0.46) in Barki sheep [30]. Similarly, positive and high direct genetic correlations between body measurement traits in Santa Ines sheep was reported [28]. Direct genetic correlation estimates varied from −0.55 (between body length and leg circumference) to 0.99 (between height at withers and height at rump) in Moghani sheep using restricted maximum likelihood [4]. Direct genetic correlation estimates between morphometric traits ranged from −0.21 (between body length and leg circumference to 0.67 (between leg circumference and heart girth). Estimates of direct genetic correlation between morphometric traits and yearling weight were positive and varied from 0.08 (between height at withers and yearling weight) to 0.52 (between heart girth and yearling weight) and selection on body length may result in negative indirect effect on height at rump, leg circumference and heart girth [22]. Similar results were reported in Makuie sheep [3]. The highest genetic correlation between yearling weight and other body measurements was between YW and SHG (0.99), followed by YW and YHG (0.87). Similar results were reported in other studies in various sheep breeds [3,22,30] and it could be explained by the fact that heart girth is a component of tissue measurements [33], whereas, other morphometric traits are part of skeletal measurements [22]. The heart girth at six months age (SHG) is most reliable body measurement among the others to predict the yearling weight and it may be taken into account while implementing genetic selection program based on biometric characters as the correlation magnitude between heart girth and yearling weight is highest. The genetic correlations among the traits at six months were higher than the correlations between the traits at nine months and yearling age. This could be explained by that selection of lambs is based on the six months body weight as there is existence of greater additive genetic variability, further, the growth and biometrical traits at six months age are mostly affected by common genes than traits at nine and twelve months age.

The estimates of phenotypic correlations between body measurements were positive and ranged from low 0.01 (SBL and YBL) to high 0.09 (NBL and NHG) and between YW and other traits the estimates ranged from 0.22 (between YW and YBL) to 0.98 (between YW and SHG). Similar results were reported in Moghani sheep [24]. From phenotype perspective, improvement of trait may occur. The estimates of environmental correlations between body measurements ranged from negative −0.001 (between YBL and SHW) to positive 0.99 (NBL and YBL) and between YWT and other body measurements the estimates ranged from low 0.21 (YW and YBL) to high 0.97 (YW and SHG). A negative environmental correlation indicates the environmental independency of these traits. Modifying the environment that affects a trait may change the character but in opposite direction. Results were contrary to the earlier reports [3,4,30].

## CONCLUSION

In summary, the direct heritability estimates obtained in the study indicated a moderate to high genetic variability for body measurements and suggested the possibility of genetic improvement through selectionBased on the correlation estimates, it is suggested that heart girth at six months of age is a reliable indicator of yearling weight from all perspectives (genetic, phenotypic, and environmental). Hence, it should be considered as a selection criterion by selecting the genetically superior lambs for chest girth at an early age which enables breeder to bring better genetic improvement in body weights at later stage. Further, it is advised to consider the negative environmental correlations while implementing the breeding decisions. Based on the correlation estimates between body measurements and yearling weights, the biometric traits may be incorporated in reliable selection index and it may have relevance in other Indian sheep breeds.

## Figures and Tables

**Table 1:** Characteristics of the data structure, summary of statistics and significance of the source of variation for post-weaning live body measurements and yearling weight in Nellore sheep

Trait	SBL^1)^	SHW^1)^	SHG^1)^	NBL^1)^	NHW^1)^	NHG^1)^	YBL^1)^	YHW^1)^	YHG^1)^	YW^1)^
Number of records	2,076	2,076	2,076	1,857	1,857	1,857	1,515	1,515	1,515	1,515
Number of animals in the pedigree	2,444	2,444	2,444	2,229	2,229	2,229	1,885	1,885	1,885	1,885
Sires with progeny records	75	75	75	75	75	75	63	63	63	63
Dams with progeny records	522	522	522	476	476	476	403	403	403	403
Animals with known paternal grand sire with progeny	1,407	1,407	1,407	1,314	1,314	1,314	908	908	908	908
Animals with known paternal grand dam with progeny	1,347	1,347	1,347	1,196	1,196	1,196	823	823	823	823
Animals with known maternal grand sire with progeny	1,214	1,214	1,214	1,044	1,044	1,044	771	771	771	771
Animals with known maternal grand dam with progeny	1,080	1,080	1,080	891	891	891	614	614	614	614
Mean	57.62	65.27	69.76	59.72	69.73	70.26	62.51	74.43	75.50	26.21
Standard deviation	6.14	7.06	7.39	6.06	5.70	5.91	5.68	5.00	4.54	5.39
Coefficient of variation (%)	10.65	10.81	10.59	10.14	8.17	8.41	9.08	6.71	6.01	20.56
Effects^2)^										
Year of lambing	^**^	^**^	^**^	^**^	^**^	^**^	^**^	^**^	^**^	^**^
Season of birth	^**^	^**^	NS	NS	^**^	^**^	^**^	NS	NS	^*^
Sex of lamb	^**^	^**^	^**^	^*^	^**^	NS	^**^	^*^	^*^	^**^
Parity of dam	^**^	NS	^**^	^**^	NS	^**^	^*^	NS	NS	NS

1) SBL, SHW, and SHG: body length, height at withers and heart girth at six months; NBL, NHW, and NHG: body length, height at withers, and heart girth at nine months; YBL, YHW, and YHG: body length, height at withers and heart girth at twelve months; YW, yearling weight.

2) Indicates the significance of the source of variation.

* p<0.05,

** p<0.01.

NS, non-significant (p>0.05).

**Table 2:** Variance components and genetic parameters for post-weaning live body measurements and yearling weight of Nellore sheep

Trait	SBL^1)^	SHW^1)^	SHG^1)^	NBL^1)^	NHW^1)^	NHG^1)^	YBL^1)^	YHW^1)^	YHG^1)^	YWT^1)^
Model	3	2	3	2	2	4	2	6	6	6
σ ^2^_a_	3.28	11.03	8.48	8.17	20.45	11.22	0.67	12.32	6.53	2.14
σ ^2^_m_	1.69	4.82	3.21	0.24	9.53	-	0.00	4.96	2.77	1.73
σ_am_	−1.73	-	−3.73	-	-	-	-	−7.34	−4.18	0.03
σ ^2^_pe_	-	-	-	-	-	0.00	-	0.69	1.24	1.18
σ ^2^_e_	22.72	20.14	29.11	25.44	23.56	20.64	30.05	14.48	14.27	14.53
σ ^2^_p_	25.96	35.99	37.06	33.61	53.56	31.85	30.72	25.11	20.63	19.62
h^2^	**0.13 (0.05)**	**0.31 (0.04)**	**0.22 (0.06)**	**0.24 (0.04)**	**0.38 (0.04)**	**0.35 (0.04)**	**0.02 (0.00)**	**0.49 (0.10)**	**0.32 (0.07)**	**0.11 (0.05)**
m^2^	0.07	0.13	0.04	0.00	0.17	-	0.00	0.20	0.13	0.09
r_am_	−0.72	-	−0.72	-	-	-	-	−0.94	−0.98	0.02
pe^2^	-	-	-	-	-	0.04	-	0.03	0.06	0.06
h^2^_t_	0.06	0.37	0.12	0.25	0.47	0.35	0.02	0.15	0.08	0.16
t_m_	0.03	0.21	0.08	0.06	0.27	0.13	0.01	0.06	0.07	0.18
Log L	−4,410.1	4,627.389	−4,756.06	3,386.095	3,644.914	3,319.865	3,354.297	3,142.987	3,020.574	−2,987.18
AIC	8,823.2	9,256.77	6,765.78	6,774.19	7,291.82	6,643.73	6,710.59	6,290.974	6,046.14	5,979.36

1) SBL, SHW, and SHG: body length, height at withers and heart girth at six months; NBL, NHW, and NHG: body length, height at withers, and heart girth at nine months; YBL, YHW, and YHG: body length, height at withers and heart girth at twelve months; YW, yearling weight.

σ^2^_a_, direct additive genetic variance; σ^2^_m_, maternal additive genetic variance; σ_am_, additive direct–maternal genetic covariance; σ^2^_pe_, maternal permanent environmental variance; σ^2^_e_, environmental variance; σ^2^_p_, phenotypic variance; h^2^, heritability; m^2^, maternal heritability; r_am_, additive direct–maternal genetic correlation; pe^2^ = σ^2^_pe_/σ^2^_p_; h^2^_t_, total heritability; t_m_, repeatability of the ewe performance; and log L, log-likelihood expressed as a deviation from the model with highest likelihood; AIC, Akaike’s information criterion.

**Table 3:** Correlations between traits yielded under bi-variate analysis

Trait^1)^		r_e_	r_a_	r_p_
SBL	SHW	0.43±0.02	0.70±0.06	0.51±0.01
SBL	SHG	0.36±0.03	0.99±0.01	0.58±0.02
SBL	NBL	0.08±0.03	0.49±0.10	0.20±0.02
SBL	NHW	0.06±0.02	0.64±0.08	0.26±0.02
SBL	NHG	0.06±0.03	0.64±0.08	0.26±0.02
SBL	YBL	−0.01±0.00	0.60±0.17	0.01±0.00
SBL	YHW	0.02±0.01	0.48±0.10	0.15±0.02
SBL	YHG	0.03±0.01	0.38±0.04	0.22±0.02
SBL	YW	−0.007±0.02	0.64±0.07	0.25±0.02
SHW	SHG	0.41±0.03	0.61±0.08	0.47±0.02
SHW	SHW	0.12±0.04	0.57±0.11	0.23±0.02
SHW	NHW	0.13±0.04	0.57±0.11	0.24±0.02
SHW	NHG	0.12±0.04	0.50±0.10	0.23±0.02
SHW	YBL	−0.001±0.003	0.27±0.04	0.14±0.02
SHW	YHW	0.06±0.04	0.85±0.09	0.25±0.02
SHW	YHG	0.10±0.03	0.95±0.08	0.27±0.02
SHW	YW	0.07±0.04	0.26±0.04	0.27±0.02
SHG	NBL	0.07±0.03	0.38±0.11	0.17±0.02
SHG	NHW	0.12±0.04	0.40±0.04	0.24±0.02
SHG	NHG	0.89±0.00	0.99±0.01	0.86±0.01
SHG	YBL	0.04±0.02	0.48±0.17	0.12±0.02
SHG	YHW	0.05±0.02	0.34±0.11	0.12±0.02
SHG	YHG	0.12±0.04	0.78±0.11	0.26±0.02
SHG	YW	0.97±0.14	0.99±0.06	0.98±0.12
NBL	NHW	0.17±0.03	0.91±0.08	0.34±0.02
NBL	NHG	0.98±0.02	0.95±0.00	0.99±0.01
NBL	YBL	0.99±0.03	0.99±0.00	0.97±0.01
NBL	YHW	0.11±0.04	0.68±0.11	0.24±0.02
NBL	YHG	0.08±0.04	0.77±0.11	0.22±0.02
NBL	YW	0.35±0.03	0.88±0.16	0.45±0.02
NHW	NHG	0.21±0.04	0.92±0.06	0.41±0.02
NHW	YBL	0.96±0.03	0.63±0.16	0.18±0.02
NHW	YHW	0.20±0.04	0.79±0.09	0.34±0.02
NHW	YHG	0.19±0.03	0.89±0.09	0.32±0.02
NHW	YW	0.20±0.04	0.40±0.05	0.27±0.02
NHG	YBL	0.05±0.02	0.40±0.17	0.11±0.03
NHG	YHW	0.16±0.04	0.56±0.11	0.26±0.02
NHG	YHG	0.19±0.04	0.72±0.10	0.30±0.02
NHG	YW	0.17±0.05	0.44±0.09	0.27±0.02
YBL	YHW	0.29±0.03	0.86±0.16	0.35±0.02
YBL	YHG	0.33±0.03	0.78±0.20	0.37±0.02
YBL	YW	0.19±0.04	0.07±0.02	0.22±0.02
YHW	YHG	0.40±0.03	0.87±0.09	0.48±0.02
YHW	YW	0.25±0.04	0.21±0.05	0.24±0.03
YHG	YW	0.40±0.03	0.87±0.09	0.48±0.02

1) SBL, SHW, and SHG: body length, height at withers and heart girth at six months; NBL, NHW, and NHG: body length, height at withers, and heart girth at nine months; YBL, YHW, and YHG: body length, height at withers and heart girth at twelve months; YW, yearling weight.

r_a_, direct genetic correlation; r_e_, residual correlation; and r_p_, phenotypic correlation.

## References

[1] (2012). Livestock Census: [internet].

[2] Mandal A, Dass G, Rout PK, Roy R (2011). Genetic parameters for direct and maternal effects on post-weaning body measurements of Muzaffarnagari sheep in India. Trop Anim Health Prod.

[3] Jafari S, Hashemi A (2014). Estimation of genetic parameters for body measurements and their association with yearling liveweight in the Makuie sheep breed. S Afr J Anim Sci.

[4] Bakhshalizadeh S, Hashemi A, Gaffari M, Jafari S, Farhadian M (2016). Estimation of genetic parameters and genetic trends for biometric traits in Moghani sheep breed. Small Rumin Res.

[5] Snyman MA, Erasmus GJ, Van Wyk JB, Olivier JJ (1995). Direct and maternal (co)variance components and heritability estimates for body weight at different ages and fleece traits in Afrino sheep. Livest Prod Sci.

[6] Abegaz S, Van Wyk JB, Olivier JJ (2005). Model comparisons and genetic and environmental parameter estimates of growth and the Kleiber ratio in Horro sheep. S Afr J Anim Sci.

[7] Kushwaha BP, Mandal A, Arora AL, Kumar R, Kumar S, Notter DR (2009). Direct and maternal (co)variance components and heritability estimates for body weights in Chokla sheep. J Anim Breed Genet.

[8] Bahreini-Behzadi MR, Aslaminejad AA, Sharifi AR, Simianer H (2014). Comparison of mathematical models for describing the growth of Baluchi sheep. J Agric Sci Technol.

[9] Singh H, Pannu U, Narula HK, Chopra A, Naharwara V, Bhakar SK (2016). Estimates of (co) variance components and genetic parameters of growth traits in Marwari sheep. J Appl Anim Res.

[10] Kumar S, Kumar V, Gangaraju G, Nath S, Thiruvenkadan AK (2017). Estimates of direct and maternal (co) variance components as well as genetic parameters of growth traits in Nellore sheep. Trop Anim Health Prod.

[11] Illa SK, Gollamoori G, Nath S (2019). Direct and maternal variance components and genetic parameters for average daily body weight gain and Kleiber ratios in Nellore sheep. Trop Anim Health Prod.

[12] Rajkumar V, Agnihotri MK, Das AK, Ramachandran N, Singh D (2010). Effect of age on carcass characteristics and meat quality of Sirohi goat kids reared under semi-intensive and intensive management systems. Indian J Anim Sci.

[13] Sasimowski E (1987). Animal breeding and production an outline.

[14] IBM Corp (2017). IBM SPSS Statistics for Windows, Version 25.0.

[15] Al-Shorepy SA (2001). Estimates of genetic parameters for direct and maternal effects on birth weight of local sheep in United Arab Emirates. Small Rumin Res.

[16] Willham RL (1972). The role of maternal effects in animal breeding: III. Biometrical aspects of maternal effects in animals. J Anim Sci.

[17] Meyer K (2007). WOMBAT—a tool for mixed model analyses in quantitative genetics by REML. J Zhejiang Univ Sci B.

[18] Akaike H (1974). A new look at the statistical model identification. IEEE Trans Automat Contr.

[19] Fourie PD, Kirton AH, Jury KE (1970). Growth and development of sheep: II. Effect of breed and sex on the growth and carcass composition of the Southdown and Romney and their cross. NZ J Agric Res.

[20] Ermias E, Rege JEO (2003). Characteristics of live animal allometric measurements associated with body fat in fat-tailed sheep. Livest Prod Sci.

[21] Alfolyan RA, Adeyinka IA, Lakpini CAM (2006). The estimation of live weight from body measurements in Yankasa sheep. Czech J Anim Sci.

[22] Salako AE (2006). Application of morphological indices in the assessment of type and function in sheep. Int J Morphol.

[23] Jafari S, Hashemi A, Darvishzadeh R, Manafiazar G (2014). Genetic parameters of live body weight, body measurements, greasy fleece weight, and reproduction traits in Makuie sheep breed. Span J Agric Res.

[24] Ghavi Hossein-Zadeh N, Ghahremani D (2018). Bayesian estimates of genetic parameters and genetic trends for morphometric traits and their relationship with yearling weight in Moghani sheep. Ital J Anim Sci.

[25] Janssens S, Vandepitte W (2004). Genetic parameters for body measurements and linear type traits in Belgian Bleu du Maine, Suffolk and Texel sheep. Small Rumin Res.

[26] Horstick A (2001). Population genetic investigation of milk production and conformation characteristics in East Frisian and black-brown dairy sheep [Doctoral dissertation].

[27] Figueiredo Filho LA, Do OAO, Sarmento JL, Santos NP, Torres TS (2016). Genetic parameters for carcass traits and body size in sheep for meat production. Trop Anim Health Prod.

[28] Oliveira EJ, El-Faro L, Freitas AP Genetic association between body measurements and weight in Santa Ines sheep.

[29] Abbasi MA, Ghafouri-Kesbi F (2011). Genetic (co) variance components for body weight and body measurements in Makooei sheep. Asian-Australas J Anim Sci.

[30] Gad SMA (2014). Estimation of genetic parameters for body measurements and their relationship with body weight in Barki lambs. J Anim Poult Prod.

[31] Falconer Douglas Scott (1996). Introduction to quantitative genetics.

[32] Matiatis N, Pollott GE (2003). The impact of data structure on genetic (co)variance components of early growth in sheep, estimated using an animal model with natural effects. J Anim Sci.

[33] Blackmore DW, Mc Gilliard LD, Lush JL (1958). Genetic relationship between body measurements at three ages in Holsteins. J Dairy Sci.

